# Veverimer, a Nonabsorbed Gastrointestinal Tract HCl Binder, Decreases Renal Ammoniagenesis and Mitigates Nephrotoxic Serum Nephritis

**DOI:** 10.34067/KID.0000000743

**Published:** 2025-03-03

**Authors:** Ali C.M. Johnson, Richard A. Zager

**Affiliations:** 1 Fred Hutch Cancer Center, Seattle, Washington; 2 Clinical Research Division, The University of Washington, Seattle, Washington; 3 Renibus Therapeutics, Dallas, Texas

**Keywords:** anti-GBM disease, chronic metabolic acidosis, complement, tubulointerstitial disease

## Abstract

**Key Points:**

- Veverimer, a nonabsorbed gastrointestinal tract HCl binder, increases bicarbonate generation, causes bicarbonaturia, and thus decreases renal ammoniagenesis.
- Decreases in ammoniagenesis can suppress kidney disease–induced alternative complement pathway activation.
- As a result of the above changes, decreases in renal injury, as induced by nephrotoxic serum injection, can occur.

**Background:**

Increased tubular ammoniagenesis is an adaptive response to progressive kidney disease, facilitating net acid excretion. However, excess ammonia production can also exacerbate kidney disease progression, in part, by activating the alternative complement cascade. Oral Na bicarbonate therapy can decrease the systemic H^+^ burden, limiting ammonia production. However, poor compliance limits bicarbonate's efficacy. Veverimer is an oral, Na^+^-free, nonabsorbed polymer that binds H^+^ within the gastrointestinal (GI) tract. This stimulates GI carbonic anhydrase-mediated bicarbonate production and systemic bicarbonate uptake. Hence, the goals of this study were to test whether GI HCl binding decreases renal tubular ammoniagenesis, to assess whether complement activation decreases, and to determine whether these changes can mitigate nephrotoxic serum (NTS) nephritis, in which complement activation may play a role.

**Methods:**

A normal diet± veverimer (4.5% w/w) was fed to normal mice for approximately 1 week. Veverimer's effect on plasma bicarbonate; blood/urinary pH; urinary ammonia excretion; and tubular H^+^ transporter, NHE3, density was assessed. Additional mice were fed the normal or veverimer diet after NTS injection. Urine protein, albumin, ammonia, C5b-9 excretion, and plasma C3a levels were measured 1 week and/or 2 weeks after NTS injection. Renal histologic changes (hematoxylin and eosin stain; C5b-9, CD45 immunohistochemistry), and selected injury mediators/biomarkers (NGAL, IL-6, MCP-1, TGF*β*1, and endothelin-1 mRNAs) were also assessed.

**Results:**

Veverimer increased plasma bicarbonate/urinary pH, reduced urinary ammonia, and decreased NHE3 in normal mice. Veverimer also reduced NTS-induced proteinuria/albuminuria, urinary ammonia, and C5b-9 excretion (by approximately 60%, 75%, and 50%, respectively). Significant reductions in NTS-induced glomerular/tubulointerstitial injury, inflammatory/profibrotic gene expression, renal C5b-9 deposition, and suppressed plasma C3a levels were observed. Oral bicarbonate also conferred protection, implicating bicarbonate's role in veverimer's beneficial effect.

**Conclusions:**

Veverimer-mediated bicarbonate generation can suppress renal ammoniagenesis and complement activation. These findings suggest a potential benefit of veverimer/bicarbonate therapy in selected complement-mediated kidney diseases.

## Visual Abstract

**Figure:**
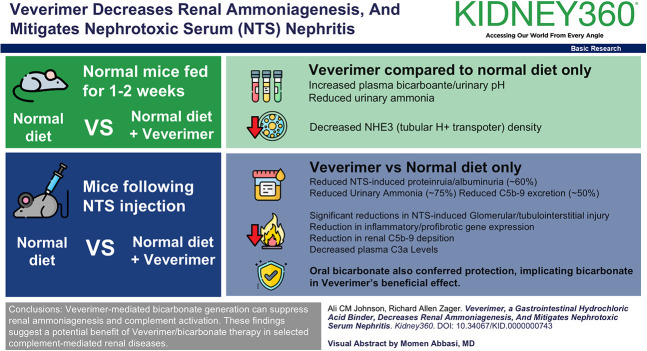

## Introduction

During the evolution of kidney disease, a loss of proximal tubules leads to a progressive reduction in total kidney ammonia production, thereby inhibiting acid excretion and potentially causing metabolic acidosis. As a compensatory response, the residual proximal tubules increase ammonia production.^1–4^ Although this adaptation helps facilitate acid excretion, high tubule ammonia levels result.^5,6^ Because ammonia can interact with C3 complement, a C3 convertase is generated, which activates the alternative complement cascade.^5–7^ This recruits inflammatory cells into the peritubular space. Combined with the generation of the C5b-9 membrane attack complex, progressive tubulointerstitial damage results.

A seminal 1985 study by Nath *et al.* confirmed the existence of these pathogenic pathways.^6^ Using the rat 5/6th nephrectomy model of CKD, a doubling of renal ammonia concentrations per functional nephron and significant peritubular C5b-9 deposition were observed.^6^ To test the pathophysiologic relevance of these findings, rats were fed Na bicarbonate for 4–6 weeks to decrease the systemic H^+^ ion burden and, thus, decrease the stimulus for increased tubule ammonia production.^6^ Bicarbonate treatment decreased tubule ammonia generation, complement activation, and C5b-9 deposition in the peritubular space. The bicarbonate diet also improved tubular function, decreased proteinuria, and mitigated progressive tubulointerstitial damage. Hence, the authors proposed the following sequence of events: renal insufficiency → H^+^ retention → compensatory increases in proximal tubule ammonia production → alternative pathway complement activation → peritubular complement deposition → inflammatory cell infiltration/C5b-9 generation → progressive tubulointerstitial damage.

These types of experimental findings have led to clinical suggestions that bicarbonate treatment may slow progression of CKD, potentially by decreasing tubular ammoniagenesis and, hence, complement activation.^5–7^ However, patient compliance with oral Na bicarbonate is poor because of its salty and bitter taste, gastrointestinal (GI) upsets, and the potential for Na retention that could worsen hypertension and heart failure.^8^ A recently proposed alternative to Na bicarbonate therapy is the oral administration of veverimer, a well-tolerated, nonabsorbed, and counter-ion/Na^+^-free polymer that specifically binds H^+^ (in the form of HCl) within the GI tract. By doing so, veverimer stimulates gastric/GI carbonic anhydrase activity, thereby generating bicarbonate, which enters the systemic circulation.^9–12^ Thus, increases in serum bicarbonate followed by urinary bicarbonate excretion (and a corresponding increase in urine pH occur. Given such changes, decreases in renal tubular ammoniagenesis, renal complement activation/deposition, and kidney disease severity could result. This study sought to test these hypotheses.

To these ends, we have assessed the effect of oral veverimer feeding on plasma bicarbonate, urinary pH, and urinary ammonia excretion in normal mice. Veverimer's effects on renal complement activation and on the severity of nephrotoxic serum (NTS) nephritis (*i.e*., antiglomerular basement nephritis) in mice were also assessed. Complement activation has been reported to be a mediator of NTS nephritis.^13–19^ Hence, this model may allow an assessment of whether veverimer can inhibit the complement cascade and associated renal damage.

## Methods

### General

Male CD-1 mice (35–45 g; Charles River Laboratories, Wilmington, MA) were used for all experiments. They were housed under standard vivarium conditions with free food (standard mouse chow) and water access. All procedures were performed under deep pentobarbital anesthesia (40–50 mg/kg body weight). Protocols complied with Institutional Animal Care and Use Committee/National Institutes of Health regulations.

### Veverimer Effects on Normal Mice

Mice were fed either standard mouse chow (*n*, 5) or the same chow containing 4.5% (w/w) veverimer (*n*, 5). Veverimer was obtained from Renibus Therapeutics, Southlake, TX. After 5–7 days on these diets, spot urine samples were obtained for measurement of pH and urinary ammonia–creatinine ratios (assay described below). The abdominal cavities were then opened, and terminal heparinized blood samples were obtained from the inferior vena cava for blood gas analysis (pH; calculated bicarbonate, cHCO_3_^−^). Then, the kidneys were resected, and cortical protein extracts were prepared and probed for NHE3, the apical membrane Na^+^/H^+^ antiporter, by ELISA (Mybiosource.com MBS2615348; San Diego, CA). The results were factored by extracted protein concentrations. The rationale for NHE3 assessment stems from observations that NHE3 density directly corresponds to the renal tubule H^+^ burden.^20^

### NTS Nephritis

A spontaneously excreted spot urine sample was collected from 12 normal mice for determination of urine pH and baseline urinary protein–creatinine concentrations (mg/mg). The mice were weighed, and then, each received a tail vein injection of 200 *µ*l of sheep NTS (Probetex, Experimental Pathology Resources, Arlington, TX). After the injections, the mice were divided equally into two groups. One group remained on a normal diet (*n*, 6). The second group was started and maintained on the 4.5% veverimer diet noted above. The veverimer diet was commenced within 30–60 minutes of NTS injection.

One week after NTS injection, a spot urine sample was collected from each mouse to assess urine pH and urine protein–creatinine ratios. Two weeks after NTS injection, the mice were deeply anesthetized, the abdominal cavities were opened, a urine sample was obtained from each mouse by gentle urinary bladder compression, and then terminal blood samples were obtained, as noted above. One kidney per mouse was transected from pole to pole and placed into 10% buffered formalin for subsequent histologic analyses (described below). Additional samples of cortical/outer medullary tissues were extracted for total RNA (RNeasy; Qiagen, Germantown, MD) and saved for subsequent measurement of injury/inflammatory/profibrotic biomarkers (NGAL mRNA, MCP-1 mRNA, IL-6 mRNA, TGF*β*1 mRNA, endothelin-1 [ET-1] mRNA; by reverse-transcription polymerase chain reaction^21,22^). Results were factored by simultaneously obtained GAPDH product. Because total renal cortical lactate dehydrogenase (LDH) concentrations provide an accurate index of total viable cortical/outer medullary tubular cell mass,^23^ its concentration per gram of tissue wet weight were also assessed.

### Blood Analyses

Terminal blood pH and plasma cHCO_3_^−^ concentrations were measured using a blood gas analyzer (ABL805 Flex; Radiometer - Brea, CA). BUN and creatinine concentrations were measured using commercial assay kits (BUN: DIUR-500; Bioassay Systems, San Francisco, CA; creatinine, BioChain, Newark, CA Z5030020).

### Urine Sample Analyses

#### Urine Protein Concentrations

Spot urine samples, obtained at baseline and at the 1- and 2-week time points, were assayed for total protein with a commercial kit (QuanTest Red, Quantimetrix 5210-12; Redondo Beach, CA). In addition, urinary albumin concentrations were determined by ELISA (Abcam ab108792, Waltham, MA). Values were factored by sample urine creatinine concentrations.

#### Urine pH

Given that veverimer generates bicarbonate in the GI tract, which then enters the circulation and ultimately urine, urine pH was tested in each of the obtained urine samples. Given that the urine sample volumes were insufficient to quantify pH with a standard pH meter, it was measured with pH-sensitive strips (pH range, 5–10; 0.1-unit gradients; EMD Millipore Supelco MQuant; 1.09533.0001; Burlington, MA).

#### Urinary Ammonia

Total urinary ammonia concentrations were measured using a commercially available fluorescence assay (Abcam, ab272538). Results were expressed as mg ammonia/mg urine creatinine.

#### Urine Membrane Attack Complex (C5b-9)

Clinical evidence indicates that complement cascade activation can be assessed by urinary C5b-9 measurement.^24,25^ Hence, urinary C5b-9 concentrations were measured 2 weeks after NTS injection±veverimer treatment (ELISA; LSBio; LS-F22262; Shirley, MA). Values were factored by urinary creatinine concentrations. Normal urine samples provided control C5b-9 concentrations.

### Plasma C3a Analysis

To further assess whether the NTS model activated the complement cascade, and if so, could veverimer suppress it, plasma samples from NTS mice±veverimer treatment were assayed for C3a (the C3 activation/cleavage product). Samples were obtained 1 and 2 weeks after NTS injection (*n*, 5–6 per group±veverimer treatment). Plasma samples from five normal mice established normal plasma C3a concentrations. C3a levels were measured by ELISA (Novus Biologicals NBP2-70037, Centennial, CO).

### Renal Histologic Analyses

Formalin-fixed renal tissues, obtained 2 weeks after NTS injection± veverimer feeding, were paraffin embedded, and 4-*μ*m sections were cut and stained with hematoxylin and eosin. The sections were scanned at 20× with Aperio ImageScope (Leica; Vista, CA; assessments performed by the Experimental Histopathology Laboratory, Fred Hutch Cancer Center, Seattle, WA). The extent of glomerular injury 2 weeks after NTS injection was compared between the veverimer versus control NTS groups by calculating the percentage of glomerular crescent formation (per 25 glomeruli counted per slide). The extent of overall tubulointerstitial histologic injury (peritubular cellular infiltrates, intraluminal debris/cast formation), was blindly graded using a semiquantitative scale of 1+ to 4+ (least to most severe injury observed).

Immunohistochemical staining for C5b-9 and CD45 were assessed as markers of complement activation and inflammatory cell infiltrates, respectively (performed by the Fred Hutch Cancer Center Experimental Histopathology Laboratory) using rabbit anti-mouse C5b-9 (Bioss bs-2673R; Woburn, MA) and anti-CD45 (Abcam ab10558), using a previously described methodology.^26^

### Mouse Anti-Sheep IgG Plasma Assay 2 Weeks after NTS Injection±Veverimer Treatment

To assess whether veverimer altered the extent of mouse anti-sheep IgG formation, and hence potential injury severity during the autologous injury phase, 2-week plasma samples from five NTS mice and five NTS/veverimer-treated mice were assayed for mouse anti-sheep IgG. Plasma samples from five normal mice served as controls. Samples were outsourced to Genesis Pharmaoptima, Portgage, Michigan, for measurement. Samples were serially diluted 1:2-1:256 and assayed in duplicate using Meso Scale Discovery Immunoassay Technology (Montreal, QC). Enhanced chemiluminescence signals were compared between the groups.

### Effect of NaHCO_3_ Diet on NTS-Induced Proteinuria

The following experiment tested the hypothesis that veverimer's protective effect was a result of bicarbonate generation. To this end, baseline (spot) urine samples were obtained from ten mice, and then the mice were injected with NTS, as noted above. They were then placed on either the normal diet (*n*, 5) or the diet supplemented with 2.1% (w/w) Na bicarbonate diet (*n*, 5), as per Nath *et al*.^6^ Seven days later, blood gas analysis, urine pH, urine protein–creatinine ratios, urine ammonia–creatinine ratios, and urine C5b-9–creatinine ratios were determined. The effect of the bicarbonate diet on the extent of NTS-induced kidney disease was assessed by measuring the degree of urine protein–creatinine ratio increases and BUN–plasma creatinine concentrations.

### Assessment of Whether Delayed Veverimer Treatment Mitigates Disease Progression

Six mice had spot urine samples collected, and then, they were injected with NTS. A repeat urine sample was collected 24 hours later, and then, the mice were divided into two equal groups that were either maintained on the normal diet or switched to the veverimer diet. One and 2 weeks later, urine protein–creatinine ratios were used to assess potential veverimer-induced benefit (N.B; as per a reviewer's comment, histology was not assessed in this set of experiments).

### Calculations and Statistics

All values are presented as means±1 SEM. Data were analyzed by either unpaired Student's *t* test or ANOVA with Tukey HSD after test. Nonparametric histologic data were analyzed by Mann–Whitney *U* test. Statistical significance was judged by a *P* value of <0.05.

## Results

### Normal Mice: Veverimer Effects on Urine pH, Blood pH, Plasma cHCO_3_^−^, Urine Ammonia, and NHE Protein Expression

The veverimer diet increased plasma cHCO_3_^−^ concentrations by approximately 2 mEq/L, matching an average approximately 2 mEq/L increments observed in prior veverimer clinical trials (see Table 1).^9,10^ The mouse cHCO_3_ increase was associated with a slight, but nonsignificant, increase in blood pH. Conversely, veverimer increased urine pH from 5.5±0.1 to 7.4±0.1 (*P* < 0.001). Veverimer also caused an approximately 75% decrease in ammonia–creatinine ratios (Table 1). A significant decrease in renal cortical Na/H^+^ antiporter (NHE3) expression was also observed (Table 1). No change in NHE3 mRNA was noted (1.14±0.06, controls; versus 1.00±0.08, veverimer; *P*, 0.2; consistent with translational versus transcriptional regulation.^27^

**Table 1:** Assessments performed on normal mouse samples± veverimer treatment

Group	Blood pH	Urine pH	P cHCO_3_^−^	Urine NH_3_/Creat	NHE3
Control	7.36±0.03	5.5±0.1	16.5±0.42	6.2±0.93	186±2.5
Veverimer	7.41±0.01	7.4±0.1	18.5±0.66	1.3±0.07	153±3.9
*P* Value	*P*, 0.18	*P*, 0.0001	*P*, 0.03	*P*, 0.0001	*P*, 0.0005

Blood/urine pH, calculated plasma bicarbonate (mEq/L), ammonia–creatinine ratios, and renal cortical NHE3 concentrations, as assessed by ELISA (ng/mg tissue protein extract). Veverimer treatment caused significant increases in urine pH and plasma bicarbonate concentrations. It also evoked an approximately 80% suppression of urinary ammonia–creatinine ratios and decreased renal cortical NHE3 protein levels. NH_3_/Creat, ammonia–creatinine; P cHCO_3_^−^, plasma bicarbonate.

### NTS Mice: Veverimer Effects on Urine pH, Urine Ammonia, Blood pH, and cHCO_3_^−^ Concentrations

Mice maintained on the normal diet and subjected to NTS injection manifested acidic urinary pH values at both the 1- and 2-week time points (see Table 2). These values did not differ from baseline urine pH levels (5.7±0.06). By contrast, the veverimer-treated mice manifested alkaline urine pH values both 1 and 2 weeks after NTS injection (Table 2). Nevertheless, veverimer did not increase either blood pH or cHCO_3_^−^ concentrations, as assessed at the terminal 2-week time point. Veverimer induced a significant, approximately 75%, reduction in urinary ammonia levels in NTS mice, consistent with findings in normal mice, as noted above.

**Table 2:** Plasma and urine analytes 1 and 2 weeks after nephrotoxic serum injection

Analyte	NTS	NTS+ Veverimer	*P* Value
Urine pH (week 1) Control, 5.7±0.06	5.7±0.03	7.8±0.25	0.0001
Urine pH (week 2) Control, 5.7±0.06	5.8±0.09	7.4±0.25	0.001
Urine NH_4_/Creat Control 6.1±0.5	4.25±0.95	1.48±0.21	0.002
P cHCO_3_^−^ (week 2) Control, 18±0.08; mEq/L	17.1±0.31	17.2±0.46	NS
Blood pH (week 2) Control, 7.4±0.03	7.48±0.01	7.45±0.01	NS
BUN (week 2) Control, 22±2; mg/dl	27±1	23±1	NS
P creat (week 2) Control, 0.3±0.03 mg/dl	0.3±0.01	0.3±0.01	NS

Urine pH, calculated plasma bicarbonate BUN (mg/dl), and plasma creatinine (mg/dl) levels determined either 1 or 2 weeks after injection of nephrotoxic serum±veverimer treatment. The *P* values were derived by comparing the nephrotoxic serum versus nephrotoxic serum+veverimer groups. Baseline urine values were obtained before nephrotoxic serum injection. Control plasma bicarbonate, pH, BUN, and plasma creatinine concentrations were determined in five normal (non–nephrotoxic serum–injected) mice. NTS, nephrotoxic serum, P cHCO_3_^−^, plasma bicarbonate; P creat, plasma creatinine.

### NTS Mice: Veverimer Effect on Urinary Total Protein and Albumin Excretion

**Figure 1:**
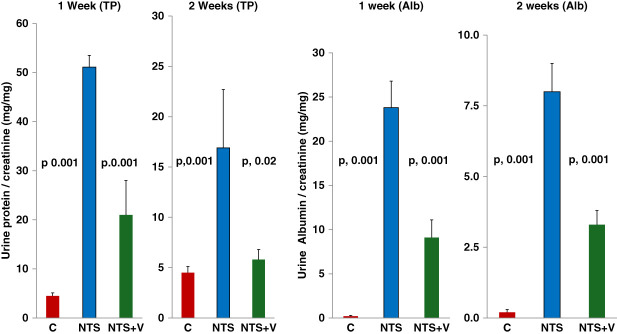
**Spot uTP–creatinine and uAlb–creatinine ratios 1 and 2 weeks after NTS injection with and without veverimer (V) administration.** Approximately ten-fold and one-hundred-fold increases in urine protein–creatinine and urine albumin–creatinine ratios, respectively, were observed at 1 week after NTS injection. At the 2-week time point, urinary protein excretion seemed to be decreasing but remained five-fold (uTP) and forty-fold (uAlb) elevated over normal values. Veverimer induced an approximately 50%–75% reduction in these values at both time points, indicating a persistent renal-protective effect (*n*, 6 per assessment, ANOVA with Tukey HSD after test). NTS, nephrotoxic serum; uAlb, urine albumin; uTP, urinary total protein.

Increases in urinary total protein and urine albumin (factored by creatinine) were seen at both 7 and 14 days after NTS injection (Figure 1). Veverimer significantly reduced urine total protein and albumin levels at both time points. Veverimer also reduced urine ammonia by approximately 65% two weeks after NTS injection (Table 2).

### Urinary C5b-9 Excretion in NTS-Treated Mice

**Figure 2:**
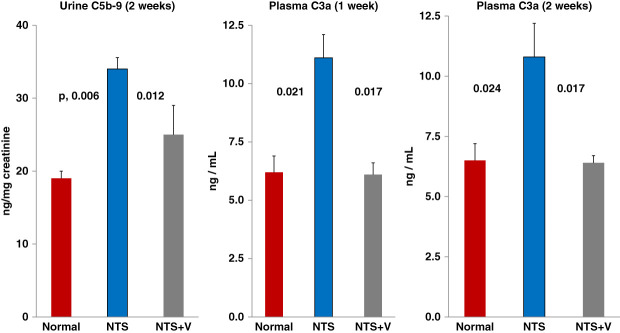
**Urinary C5b-9–creatinine ratios in mice 2 weeks after NTS injection and plasma C3a concentrations 1 and 2 weeks after NTS injection with and without veverimer (V) administration.** Two weeks after NTS injection, an approximately 80% increase in urine C5b-9–creatinine ratios was observed (compared with baseline values). Further indicating complement activation, NTS caused significant increases in plasma C3a concentrations at both the 1- and 2-week time points. When NTS-injected mice were treated with veverimer, both urinary C5b-9 ratios and plasma C3a levels were normalized, with values not significantly differing from those observed in normal urine samples (*n*, 6 per assessment, ANOVA with Tukey HSD after test).

NTS injection increased urine C5b-9–creatinine ratios by approximately 50% over control values (see Figure 2). Veverimer significantly reduced these urinary C5b-9 increases, essentially returning them to normal levels (NS versus baseline values).

### Plasma C3a Assessments

Approximately 70%–80% increases in plasma C3a levels were observed 1 and 2 weeks after NTS injection, versus normal plasma values (Figure 2). Veverimer treatment completely suppressed these NTS-mediated C3a increments (NS versus normal values).

### Renal Cortical Injury and Inflammatory mRNA Markers after NTS Injection±Veverimer

NTS injection significantly increased each of the assessed mRNAs, consistent with injury (NGAL), inflammation (MCP-1, IL-6, and ET-1), and fibrosis (TGF*β*1) (Table 3). In each instance, treatment with veverimer significantly reduced these NTS-induced gene responses. NTS injection caused an approximately 25% decrease in renal cortical/outer medullary LDH content, consistent with a decrease in viable tubule cell mass.^23^ This LDH loss was significantly (*P*, 0.01) blunted by veverimer treatment (Table 3).

**Table 3:** Veverimer: Renal tissue analyses of injury mediators and markers

Analyte	Normal	NTS	NTS+Veverimer	*P* Value
NGAL mRNA	0.1±0.001	3.5±1.1	0.8±0.2	0.03
MCP-1 mRNA	0.03±0.01	1.43±0.24	0.98±0.19	0.06
IL-6 mRNA	0.14±0.01	1.94±0.32	1.08±0.10	0.01
TGF*β*1 mRNA	0.68±0.04	1.71±0.18	1.27±0.10	0.05
ET-1 mRNA	0.18±0.02	0.70±0.11	0.40±0.04	0.03
LDH, units/g	1000±12^a^	747±10	882±20	0.01

Renal cortical mRNA and tissue lactate dehydrogenase levels in normal mice and in mice 2 weeks after nephrotoxic serum injection±veverimer treatment. Nephrotoxic serum nephritis caused a significant rise in each of the mRNAs compared with values seen in normal mice (each *P* < 0.001). The *P* values presented in the right-hand column represent the comparisons of the nephrotoxic serum versus nephrotoxic serum/veverimer-treated mice. In each instance, veverimer significantly suppressed the nephrotoxic serum-induced mRNA increases. Statistics using ANOVA/Tukey HSD after test. Veverimer-mediated protection was also indicated by modest, but significant, preservation of renal cortical lactate dehydrogenase content (nephrotoxic serum versus nephrotoxic serum+veverimer; unpaired *t* test). ET-1, endothelin-1; LDH, lactate dehydrogenase; NTS, nephrotoxic serum.

a The control lactate dehydrogenase values represent historical controls and are shown as a reference.

### Renal Histology

**Figure 3:**
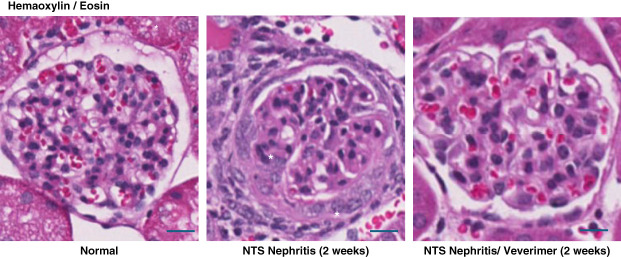
**Glomerular histology 2 weeks after NTS injection with and without concomitant veverimer treatment (H&E staining).** NTS induced acute GN with crescent (*) formation and mesangial expansion. Veverimer significantly reduced glomerular injury, as evidenced by an absence of crescent formation (consistent with a 50%–60% decrease in proteinuria as shown in Figure 1). Scale bars, 50 *µ*M. H&E, hematoxylin and eosin.

**Figure 4:**
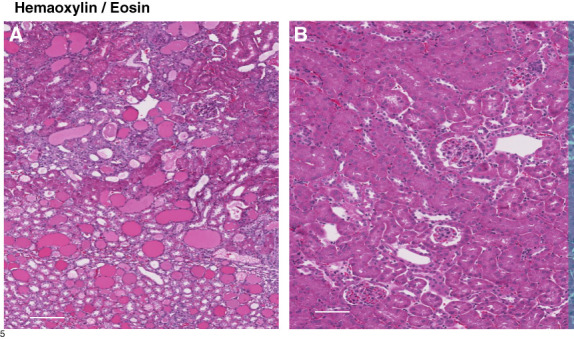
**NTS-induced cast formation, tubular injury, and interstitial nephritis (H&E staining).** As shown on the left(A), NTS injection resulted in extensive cast formation, tubular degeneration, cellular debris sloughing into tubular lumina (black arrows), and interstitial inflammation. These changes were almost completely prevented by veverimer treatment (B). Scale bars, 200 *µ*M.

**Figure 5:**
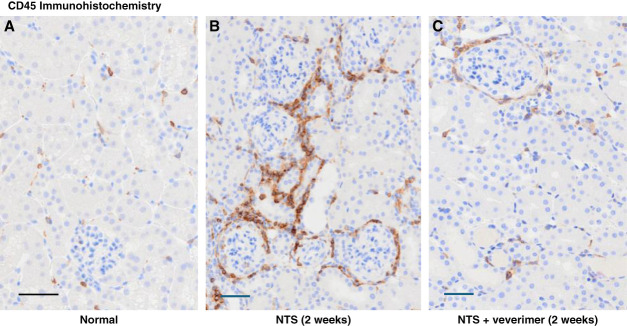
**C45 staining of renal tissues from a normal mouse and from NTS-injected mice without or with veverimer treatment.** Only occasional CD45^+^ cells were observed in normal renal tissue (A). By contrast, increased tubulointerstitial and periglomerular CD45^+^ cell accumulation was observed 2 weeks after NTS injection (B). Veverimer treatment decreased peritubular CD45^+^ cell accumulation, as depicted in (C). Scale bars, 100 *µ*M.

**Figure 6:**
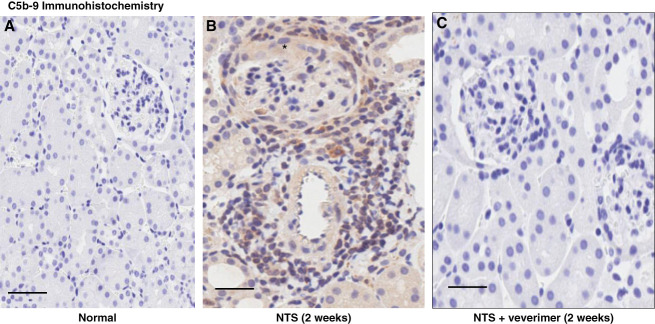
**C5b-9 deposition, assessed by immunohistochemistry, 2 weeks after NTS injection with and without veverimer treatment.** No evidence of C5b-9 deposition was seen in normal kidney sections (A). NTS caused heavy C5b-9 deposition throughout the histologic sections, as denoted by beige/light brown C5b-9 staining. This was accompanied by histologic evidence of tubulointerstitial nephritis with marked inflammatory cell infiltrates. A glomerular crescent is depicted (*). Conversely, C5b-9 deposits were not observed with veverimer treatment (B). Scale bars, 85 *µ*M.

Glomerular crescent formation was seen in 19%±3% of glomeruli 2 weeks after NTS injection without veverimer (Figure 3). Conversely, no evidence of crescent formation was noted in sections obtained from NTS/veverimer-treated mice (*P*, 0.01). NTS induced tubular atrophy, sloughing of tubular cell debris, cast formation, and tubulointerstitial/periglomerular infiltrates of CD45^+^ cells (Figures 4–6). The overall extent of these tubulointerstitial changes was significantly reduced by veverimer treatment (3.4±0.2 versus 1.8±0.2; *P* < 0.01).

C5b-9 deposition was not apparent in normal kidney sections (Figure 6A). Conversely, heavy tubulointerstitial C5b-9 deposition, denoted by light brown tissue staining, was observed 2 weeks after NTS injection (Figure 6B). Inflammatory cell infiltrates were again observed. By contrast, C5b-9 staining and tubulointerstitial infiltrates were virtually absent in the NTS nephritis mice which had been treated with veverimer (Figure 6C).

### Body Weights

Mouse body weights before NTS injection were 39.7±1 g. Two weeks after NTS injection, neither weight gain or loss nor a difference between the veverimer-treated and non–veverimer-treated mice was observed (39.7±1.6 versus 39.4±1.4 g, respectively).

### Mouse Anti-Sheep IgG Titers 2 Weeks after NTS Injection

Strong mouse anti-sheep IgG enhanced chemiluminescence signals were observed in all NTS-treated mice. Serial plasma dilutions produced an approximate successive halving of the antibody signal from the 1:32 to 1:256 dilutions. At each dilution tested, a slightly stronger signal was observed in the veverimer NTS versus control NTS samples (Table 4). Normal mouse plasma had no discernible signal.

**Table 4:** Mouse anti-sheep IgG antibody levels 2 weeks after nephrotoxic serum injection

Plasma Dilution	1:32	1:64	1:128	1:256
NTS, controls	3.65±0.29	3.2±0.13	3.0±0.16	2.8±0.3
NTS+veverimer	4.12±0.27	4.0±0.28	3.6±0.30	3.2±0.3
*P* value	0.25	0.046	0.64	0.20

Plasma samples were serially diluted from 1:2 to 1:256. The enhanced chemiluminescence antibody signals were converted to log base l0. Starting at serial dilution 1:32, approximate halving of the enhanced chemiluminescence signals commenced. At all dilutions, the enhanced chemiluminescence antibody signals were higher in plasma samples from veverimer-treated animals. Assay plates were coated with normal sheep IgG (R&D Systems, cat. 5-001-A; I *μ*g/ml), *i.e*., capture antigen. Assay buffer and 1% casein blocker were obtained from Thermo Fisher (cat. 37528). After solution coating, 25-*µ*l samples of serial plasma dilutions (from 1:2 to 1:256) were added per well. The plates were incubated with shaking (600 rpm)×2 hours and then washed. Goat anti-mouse IgG (Meso Scale Discovery cat. R32AC-1, labeled with caged ruthenium) was added, followed by washing. A read buffer (Meso Scale Discovery) was applied, and the plates were read on an Meso Scale Discovery (SQ120) instrument. Samples were run in duplicates. NTS, nephrotoxic serum.

### Effect of NaHCO_3_ Diet on NTS Nephritis

The Na bicarbonate diet recapitulated veverimer's protective effect, as assessed by reductions in proteinuria 1 week after NTS injection (50% decrease with bicarbonate versus 60% decrease with veverimer; see Table 5). As expected, bicarbonate therapy increased urine pH (from 5.7 to 8.4; Table 5). Bicarbonate administration also raised the plasma bicarbonate concentration from 16 to 20 mEq/L, but without significantly affecting blood pH (presumably reflective of respiratory compensation). Bicarbonate therapy decreased urinary ammonia by approximately 90% (NTS versus NTS + veverimer). NTS caused an approximately 80% increase in urinary C5b-9–creatinine ratios (Table 5; *P* < 0.005), which was totally blocked by Na bicarbonate therapy (*P* < 0.001; Table 5).

**Table 5:** Nephrotoxic serum nephritis with and without Na bicarbonate therapy

Analyte	Control	NTS	NTS+NaHCO_3_	*P* Value
U prot/Cr	4.8±0.6	59±11	27±3.4	0.03
U C5b-9/Cr	20±0.92	38±4.6	18±2.1	<0.001
Arterial pH	7.4±0.03	7.43±0.03	7.40±0.01	NS
Urine pH	5.7±0.06	5.7±0.06	8.4±0.18	0.001
P cHCO_3_^−^	18±0.08	16±0.26	20.0±0.69	0.008
BUN	22±1	33±2	27±1	0.023
P Cr	0.35±0.01	0.35±0.02	0.35±0.02	NS
Urine NH_3_/Cr	9.0±1.81	3.87±0.56	0.37±0.05	<0.001

Urine and plasma analytes in control mice and mice that were subjected to nephrotoxic serum without or with concomitant Na bicarbonate therapy (assessments performed 1 week after nephrotoxic serum injection). Nephrotoxic serum caused large increases in the urine protein–creatinine and urine C5b-9–Cr ratios (*P* < 0.01; not depicted). Each of these increases was significantly reduced by bicarbonate therapy. Nephrotoxic serum, by itself, decreased urinary ammonia–creatinine concentrations compared with values seen in control mice (see text; *P* < 0.01). Bicarbonate further depressed urinary ammonia–creatinine ratios in nephrotoxic serum–injected animals. As expected, bicarbonate increased urine pH and plasma bicarbonate concentrations. The depicted *P* values reflect the nephrotoxic serum versus nephrotoxic serum+bicarbonate treatment groups. Nephrotoxic serum slightly increased the BUN, but not the plasma creatinine concentrations. Cr, creatinine; NH_3_, ammonia; NTS, nephrotoxic serum; P cHCO_3_^−^, plasma bicarbonate; P Cr, plasma creatinine; U prot, urine protein.

### Delayed Veverimer Treatment Mitigates Disease Progression

Within 24 hours of NTS injection, an approximately thirty-fold increase in urinary total protein–creatinine ratios was observed (controls, 4.1±0.7; NTS, 113±6.4; *P* < 0.001). Indeed, proteinuria was greater 24 hours after NTS injection versus at any other time point, as reported above. Despite starting veverimer 24 hours after NTS injection, at the height of proteinuria, a beneficial effect was still observed, as gauged by a significant decrease in urine total protein–creatinine ratios at both 8 and 14 days after NTS injection (8 days: 62±7 versus 39±2; *P*, 0.041; 14 days: 30±5.6 versus 8.6±2.7; *P*, 0.026; control diet, veverimer diet, respectively).

## Discussion

The results of previous clinical trials support the concept that veverimer increases GI tract H^+^ (HCl) binding, thereby stimulating GI carbonic anhydrase-mediated bicarbonate production. With bicarbonate entry into the systemic circulation, modest, but statistically significant, plasma bicarbonate elevations (approximately 2-4 mEq/L) have resulted.^9,10^ This experimental study has recapitulated this result in mice, given that comparable plasma bicarbonate increases (approximately 2-4 mEq/L) were observed. In addition, veverimer feeding increased urinary pH from approximately 5.5 to approximately 7.5. This was almost certainly due to decreased tubular bicarbonate reabsorption of the elevated filtered bicarbonate load.

Given that the systemic H^+^ burden is the prime stimulus for tubule ammonia generation, we tested whether veverimer-mediated bicarbonate production can decrease tubule ammoniagenesis. This was the case, given that veverimer administration caused approximately 75% reductions in the ammonia–creatinine ratios in both normal mice and mice with NTS nephritis. Veverimer also suppressed renal cortical NHE3 expression in normal mice. Given that both ammoniagenesis and tubule NHE3 abundance directly correlate with the tubule H^+^ burden,^20,27,28^ these complementary findings support the concept that veverimer-induced bicarbonate generation decreased the tubule H^+^ load. It is noteworthy that veverimer is a nonabsorbed polymer, thereby excluding a direct veverimer effect on either tubule NHE3 expression or ammonia production. That Na bicarbonate administration also decreased ammoniagenesis further supports the concept that bicarbonate generation, and not veverimer, *per se*, mediated veverimer's renal actions.

As previously noted, in the presence of renal injury, tubule ammonia production per functional nephron increases.^6^ Although this helps to maintain net acid excretion, it also represents a maladaptive response that can perpetuate renal damage. In part, this is believed to be due to ammonia-C3 binding, thereby generating a C3 convertase (*e.g*., refs. 6 and 7). The latter triggers downstream activation of the alternative complement cascade, culminating in tubulointerstitial inflammatory cell infiltrates, cytokine pathway activation, endothelin production, and the generation of the C5b-9 membrane attack complex (*e.g*., refs. 6 and 7). These considerations led us to assess whether veverimer, using its ability to suppress ammoniagenesis, could mitigate complement activation and injury in the NTS nephritis model. As expected, large increases in proteinuria and albuminuria were noted both 1 and 2 weeks after NTS injection. At both time points, veverimer suppressed these urinary protein increases by approximately 50%–60%, indicating renal protection. The latter was confirmed by histologic analyses, given that significant reductions in both glomerular and tubulointerstitial injuries were observed. Consistent with these findings, veverimer also suppressed proinflammatory (IL-6, MCP-1, ET-1) and profibrotic (TGF*β*1) cytokine gene expression and induced a 75% decrease in NGAL mRNA, a tubular injury biomarker. Hence, each of the above findings, and a relative preservation of cortical LDH, clearly indicates a marked veverimer-mediated renal-protective effect. It is noteworthy that veverimer's beneficial effect could be expressed even when it was administered 24 hours after NTS injection, a time at which heavy proteinuria was already expressed. This indicates a therapeutic, or renal rescue, effect.

To test the concept that these changes may have been due, at least in part, to suppressed complement activation, tubulointerstitial C5b-9 deposition was probed by immunohistochemistry. As shown in Figure 6, NTS activated the complement cascade, as evidenced by heavy renal C5b-9 deposition, particularly within the tubulointerstitial space. This change was virtually completely suppressed in veverimer-treated mice. Given that urinary C5b-9 concentrations can serve as a semiquantitative marker of renal complement activation (*e.g*., in IgA nephropathy; refs. 24 and 25), C5b-9 concentrations were measured in 2-week post-NTS urine samples. NTS-treated mice demonstrated a doubling of urinary C5b-9 concentrations, a response which was blocked by veverimer treatment. To further test for NTS-induced complement activation, plasma levels of the C3 activation fragment C3a were measured. That a near doubling of plasma C3a concentrations was observed both 1 and 2 weeks after NTS injection and that these increases were completely suppressed by veverimer treatment support the above C3b-9 immunohistochemistry data. Hence, these findings support the concept that veverimer suppressed activation of the complement cascade. The latter was almost certainly due to veverimer-mediated bicarbonate generation, given that oral bicarbonate blocked urinary C5b-9 increases and suppressed proteinuria after NTS injection.

Although the above data clearly indicate that veverimer suppressed complement activation, it remains to be proven that the observed renal protection was predominantly due to complement pathway inhibition, *per se*. In this regard, ammonia generation has been suggested to evoke tissue injury by both complement dependent and independent^29–31^ pathways. As examples of the latter, excess ammonia may exert adverse effects on gene expression, apoptosis, immune responsiveness, and metabolic and innate immune signaling pathways.^31^ Further suggesting that factors in addition to complement activation may have been at play is that complement depletion, induced in rodents by cobra venom factor injection or gene C3 and C4 knockout, has conferred only partial protection against NTS (*e.g*., refs. 15–19). Hence, although the current studies clearly indicate that veverimer suppressed ammoniagenesis, complement activation, and disease severity, establishing a direct mechanistic link between these results require further study.

Another outstanding issue is that NTS nephritis has two injury phases: the heterologous injury phase (induced by sheep anti-glomerular basement membrane antibody injection), followed within approximately 4–7 days by the autologous injury phase (mediated by mouse anti-sheep antibody production). We cannot state during which (or potentially in both) injury phase veverimer exerts its protective effects. We can state the degree of autologous phase antibody production was not affected by veverimer treatment, given the results of the mouse anti-sheep antibody titer assessments. Future detailed kinetics studies in which different injury pathways are examined at multiple time points in the presence and absence of veverimer might be helpful in resolving this complex issue.

In conclusion, this study has demonstrated that veverimer administration to mice results in bicarbonate generation, bicarbonaturia, decreased renal ammoniagenesis, suppression of the complement cascade, and reduced severity of NTS-induced nephritis. Veverimer-mediated bicarbonate generation seems to be the proximate cause of these outcomes. However, the operative downstream mechanistic links between decreased ammoniagenesis, complement pathway suppression, and NTS disease reduction and the timing at which this protection is evoked remain to be more completely defined.

## Data Availability

All data are included in the manuscript and/or supporting information. Partial restrictions to the data and/or materials apply. Deidentified data are available upon reasonable request to the corresponding author.
